# The Intriguing Contribution of Hippocampal Long-Term Depression to Spatial Learning and Long-Term Memory

**DOI:** 10.3389/fnbeh.2022.806356

**Published:** 2022-04-25

**Authors:** Martin Stacho, Denise Manahan-Vaughan

**Affiliations:** Department of Neurophysiology, Medical Faculty, Ruhr University Bochum, Bochum, Germany

**Keywords:** LTD, LTP, hippocampus, spatial learning and memory, rodent

## Abstract

Long-term potentiation (LTP) and long-term depression (LTD) comprise the principal cellular mechanisms that fulfill established criteria for the physiological correlates of learning and memory. Traditionally LTP, that increases synaptic weights, has been ascribed a prominent role in learning and memory whereas LTD, that decreases them, has often been relegated to the category of “counterpart to LTP” that serves to prevent saturation of synapses. In contradiction of these assumptions, studies over the last several years have provided functional evidence for distinct roles of LTD in specific aspects of hippocampus-dependent associative learning and information encoding. Furthermore, evidence of the experience-dependent “pruning” of excitatory synapses, the majority of which are located on dendritic spines, by means of LTD has been provided. In addition, reports exist of the temporal and physical restriction of LTP in dendritic compartments by means of LTD. Here, we discuss the role of LTD and LTP in experience-dependent information encoding based on empirical evidence derived from conjoint behavioral and electrophysiological studies conducted in behaving rodents. We pinpoint the close interrelation between structural modifications of dendritic spines and the occurrence of LTP and LTD. We report on findings that support that whereas LTP serves to acquire the general scheme of a spatial representation, LTD enables retention of content details. We argue that LTD contributes to learning by engaging in a functional interplay with LTP, rather than serving as its simple counterpart, or negator. We propose that similar spatial experiences that share elements of neuronal representations can be modified by means of LTD to enable pattern separation. Therewith, LTD plays a crucial role in the disambiguation of similar spatial representations and the prevention of generalization.

## Introduction

Hippocampal long-term potentiation (LTP) and long-term depression (LTD) were first described in the 1970s (Bliss and Lomo, 1973; Alger and Teyler, 1976). Comprising a persistent, input-specific increase, or decrease of synaptic strength, respectively, LTP and LTD were initially ascribed roles in information encoding and deletion related to memory acquisition and forgetting (Tsumoto, 1993). Others have argued that LTD silences the memory engram through synaptic weakening (Nabavi et al., 2014; Basu and Siegelbaum, 2015; Luchkina and Bolshakov, 2019; Josselyn and Tonegawa, 2020). Evidence from studies conducted in freely behaving rodents during learning events, indicate, however, that LTP and LTD support different kinds of information storage, and that input-specific information storage can be differentiated according to both the afferent input and the hippocampal subfield involved (Table 1). Furthermore, induction of hippocampal LTP and LTD results in nuclear immediate early gene (IEG) mRNA expression in hippocampal neurons, albeit in distinctly different distributions (Hoang et al., 2021) that correspond to hippocampal gene encoding in response to spatial learning events (Hoang et al., 2018).

**TABLE 1:** Overview of changes in synaptic weights triggered in the hippocampus by specific components of spatial, learning in freely behaving rodents.

Synaptic plasticity facilitated by:	Sc-CA1	AC-CA3	MF-CA3	PP-DG
novel exposure to a global change in space involving an allocentric shift	LTP^1,2,3,4^	LTP^5^	LTP^5^	LTP^4,9^
exposure to a novel spatial configuration of discretely located features within a known environment	LTD^2,4,6,7,8, 10,11^*	LTD^5^	No change^5^	No change^4^
exposure to a novel spatial configuration of landmarks/ orientation-relevant features within a known environment	No change^4^	No change^5^	LTD^5^	LTD^4^

Numbers (signifying publications) marked with an asterisk (*) refer to studies in adult mice, all other studies were conducted in the dorsal hippocampus of adult rats.

*In the experiments with exposure to novel objects and spatial configurations, the animals were not required to learn any particular tasks but rather simply explored a novel environment created by an empty hole board, a hole board containing unfamiliar objects, or new object configurations (e.g., items in holeboard holes or spatial configurations of landmark features).*

*Schaffer collateral- CA1 synapses: Sc-CA1, commissural associational- CA3 synapses; AC-CA3; mossy fiber-CA3 synapses: MF-CA3; perforant path-dentate gyrus synapses: PP-DG.*

*^1^Manahan-Vaughan and Braunewell (1999); ^2^Kemp and Manahan-Vaughan (2004); ^3^Straube et al. (2003); ^4^Kemp and Manahan-Vaughan (2008b); ^5^Hagena and Manahan-Vaughan (2011); ^6^Lemon and Manahan-Vaughan (2012); ^7^André and Manahan-Vaughan (2013); ^8^Dietz and Manahan-Vaughan (2017), ^9^Hansen and Manahan-Vaughan (2015); ^10^Kemp and Manahan-Vaughan (2012); and ^11^Goh and Manahan-Vaughan (2013a; 2013b).*

A functional role for LTP has been described in the acquisition of conditioned fear memory (Ramirez et al., 2013; Luchkina and Bolshakov, 2019; Josselyn and Tonegawa, 2020), in the acquisition of information about novel space (Kemp and Manahan-Vaughan, 2004), or the gaining of knowledge about the allocentric context of space (Straube et al., 2003; Manahan-Vaughan, 2017). On the other hand, LTD has been implicated in the acquisition of information about novel item configurations (Manahan-Vaughan and Braunewell, 1999; Goh and Manahan-Vaughan, 2013c) spatial information updating (Kemp and Manahan-Vaughan, 2004, 2008a,b) and spatial memory consolidation (Ge et al., 2010; An and Sun, 2018). Furthermore, animals with impaired LTD show deficits in long-term, but not short-term, contextual fear memory (Liu et al., 2014), and either a complete inability to succeed in the Morris Water Maze task (Etkin et al., 2006; Rocchetti et al., 2015), or a deficit in reversal learning when the hidden platform is changed to another quadrant (Nicholls et al., 2008; Kim et al., 2011; Mills et al., 2014). Together with an impaired ability to habituate to novel space and objects (Etkin et al., 2006), this indicates that when LTD is impaired, animals are unable to form a proper detailed representation of space, or modify the representation. Indeed, hippocampal LTD is tightly associated with the *de novo* acquisition of knowledge of spatial content (Kemp and Manahan-Vaughan, 2008b; Hagena et al., 2016; Manahan-Vaughan, 2018a) or the *updating* of spatial content information (Kemp and Manahan-Vaughan, 2004; Goh and Manahan-Vaughan, 2013c; Manahan-Vaughan, 2018a). We have proposed in the past that LTP and LTD work together to create a memory “engram” comprised of a neuronal network in which LTP and LTD, of designated synapses, serves to create a unique and discriminable representation of associative experience (Kemp and Manahan-Vaughan, 2007; Manahan-Vaughan, 2017, 2018a,b). Recent evidence suggests that both the structural modifications of synapses, and the temporal and physical restriction of LTP by LTD in dendritic subcompartments may support this process. In this review article, we highlight the role of LTD in spatial content representation and long-term memory and describe how it also leads to information encoding by means of nuclear immediate early gene expression. We then discuss structural modifications of dendritic spines and describe the interrelationship between structural and functional synaptic plasticity. Finally, we describe reports on the physiological interactions of LTD with LTP in the dendritic domain. We propose that LTD creates a robust neuronal representation of spatial content by means of eliminating weakly potentiated synapses and by dictating temporal constraints and the dendritic distribution of LTP. By this means LTD not only enables spatial content encoding and updating, but also supports pattern separation and subverts experience generalization under circumstances where similar experiences are represented by shared neuronal elements.

## Experimental Evidence for a Role for Long-Term Depression in Learning and Memory

**FIGURE 1:**
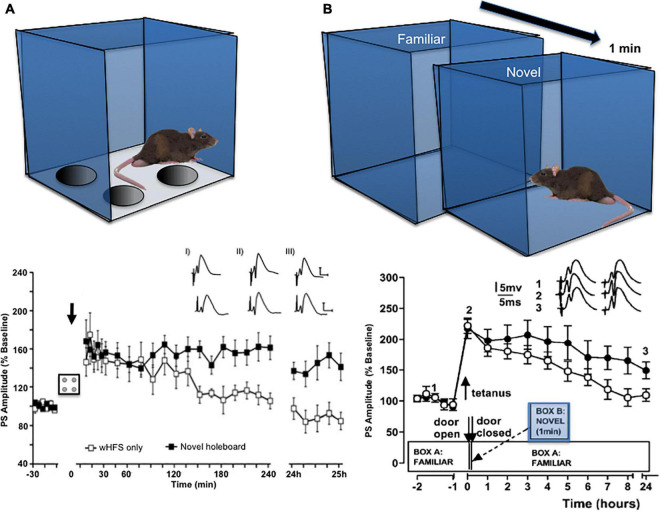
Exposure to novel allocentric space facilitates the induction of hippocampal LTP. **(A)** Insertion of a novel holeboard into a familiar environment (Ai) promotes the expression of LTP in the hippocampus of rats. In the graph shown, weak high frequency stimulation (wHFS) of perforant path (pp) synapses to the dentate gyrus (DG) triggers short-term potentiation (STP) that lasts for maximally 2 h (unfilled squares). Combination of wHFS (arrow) during exposure to a novel holeboard transforms STP into LTP that lasts for over 24 h (black squares). Inset: analogs show examples of field excitatory post synaptic potentials (fEPSPs) recorded prior to wHFS (iI), 5 min (ii) and 24 h(ii) after wHFS in animals that received wHFS only (top row) and animals that received wHFS during holeboard exploration (bottom row). Scale bar, vertical: 5 mV, horizontal, 5 ms. From Kemp and Manahan-Vaughan, 2008b. **(B)** Migration from a familiar environment to an adjacent novel environment 2 min after tetanic afferent stimulation (comprising exploration for 1 min, followed by a return to the familiar environment) promotes the expression of LTP at pp-DG synapses in rats. The graph describes how application of the tetanus alone resulted in STP (unfilled circles), compared to when the tetanus was applied in conjunction with novel environmental exposure (filled circles). Inset: analogs show examples of fEPSPs recorded at the time point signified by the digits in animals that received tetanus only (left) and that received tetanus followed by novel environment exploration (right). The graph shown **(B)** is from Straube et al., 2003, with permission.

Causal proof that synaptic plasticity enables learning is not easy to obtain and it is still being discussed that other mechanisms may play a role (Titley et al., 2017; Abraham et al., 2019). However, studies that examined the expression of hippocampal synaptic plasticity during, and as a result of, spatial learning events have shown that LTP emerges when a rat is repeatedly shown a spatial environment in which its allocentric relationship to distal cues is adjusted (Straube et al., 2003; Figure 1A). LTP is also facilitated in an input-specific manner in different synaptic subcompartments of the hippocampus such as the perforant path-dentate gyrus synapse, the mossy fiber-CA3 synapse, commissural associational–CA3 synapse and the Schaffer collateral-CA1 synapse, when a rat is exposed to a global allocentric change in its spatial environment for the first time (e.g., introduction of a novel holeboard into a familiar environment) (Kemp and Manahan-Vaughan, 2004, 2008b; Hagena and Manahan-Vaughan, 2011; Figure 1B). The fact that synapses are potentiated in a *distributed* manner through the hippocampus by a novel allocentric experience suggests that the initial step in the creation of a spatial representation is the selection of a synaptic network, by means of LTP. Thus, LTP seems to be the “first-responder” event in the hippocampus that occurs in a widespread, albeit input-specific manner, when an animal is confronted with a novel spatial environment, or with salient allocentric changes of a known spatial environment. This property aligns with reports that LTP can be induced with just a single afferent volley (Gustafsson and Wigstrom, 1986; Gustafsson et al., 1987), whereas LTD requires minutes to manifest and stabilize (Dudek and Bear, 1993; Manahan-Vaughan, 1997). Scrutiny of the behavioral learning circumstances, in which LTD emerges, have revealed a more heterogeneous role compared to that observed for LTP. Thus, LTD is associated with very specific forms or components of spatial learning and its expression is localized to discrete subcompartments of the hippocampus (Kemp and Manahan-Vaughan, 2008b; Hagena and Manahan-Vaughan, 2011).

### The Link Between Long-Term Depression and Recognition Memory

The first hint that hippocampal LTD may play a role in information encoding was provided by a study that described the emergence of LTD when afferent stimulation of Schaffer collateral fibers (to induce weak synaptic depression), coupled with exposure of rats to novel objects, transformed short-term depression (STD) into LTD that lasted for days in the hippocampal CA1 region (Manahan-Vaughan and Braunewell, 1999). Subsequent studies in mice revealed a similar relationship: Test pulse stimulation of Schaffer collateral fibers coupled with exposure to novel objects results in LTD in the CA1 region (Goh and Manahan-Vaughan, 2013c). The facilitation of LTD by novel item exploration recruits protein synthesis (Dong et al., 2012; Kemp et al., 2013), a property that has been proposed as a criterion for the qualification of a cellular process as a memory mechanism (Martin et al., 2000). Transgenic, or pharmacological, manipulation of proteins relevant for synaptic plasticity provided additional mechanistic insights into this process. For example, genetic deletion of serum response factor (SRF) (Etkin et al., 2006), or manipulation of neuregulin-signaling (Ledonne et al., 2018), impairs both hippocampal LTD and object recognition memory. Moreover, SPIN90-knockout mice exhibit deficits in hippocampal LTD and object recognition memory (Kim et al., 2017), Bcl-2 associated protein (Bax) knockout mice exhibit deficits in long-term, but not short-term memory, that are accompanied by LTD impairments (Liu et al., 2014), and inhibition of LTD through antagonism of plasticity-related neurotransmitter receptors also prevents object recognition memory (Goh and Manahan-Vaughan, 2013a,b). Here, it is important to point out that a clear delineation has been proposed between the role of the perirhinal cortex in item recognition *per se* (Aggleton et al., 2010) and the role of the hippocampus in item recognition at the level of item-place recognition and spatial elements of item recognition memory (Wan et al., 1999; Brown and Aggleton, 2001). Although in the former case, LTD in the perirhinal cortex is likely to be involved (Griffiths et al., 2008), closer scrutiny of the relationship between hippocampal LTD and object recognition memory has revealed that it is not the identity of the object itself, but rather the relationship of the object to its location in space that is encoded by hippocampal LTD (Kemp and Manahan-Vaughan, 2004).

### The Link Between Long-Term Depression and Item–Place Learning

**FIGURE 2:**
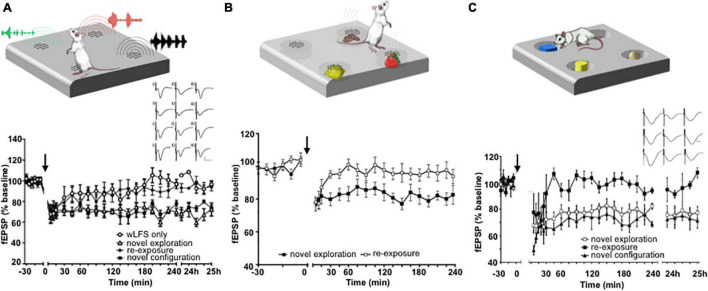
Exposure to item-place experience facilitates the expression of hippocampal LTD. **(A)** Exploration of novel spatial configurations of auditory items (top) promotes the expression of hippocampal LTD. Graph: weak low frequency stimulation (wLFS, 1 Hz 600 pulses) applied to Schaffer collateral CA1 (SC-CA1) synapses results in short-term depression (STD) in freely behaving rats that lasts for ca. 30 min. Combination of wLFS with novel exploration of audiospatial configurations results in the facilitation of STD into LTD. A subsequent re-exposure to the same items in the same locations at least 7 days after the first exposure during wLFS results in STD. But combining wLFS with the exposure to a novel configuration of the same auditory items results in LTD that lasts for over 24 h. Inset: analogs show examples of field excitatory post synaptic potentials (fEPSPs) recorded prior to wLFS (i), 5 min (ii) and 24 h (iii) after wLFS in animals that received wLFS only (top row), animals that engaged in novel audiospatial cue exploration (2nd row), animals that experienced re-exposure to the cues (3rd row) and animals that were exposed to a novel audiospatial cue configuration (bottom row) Scale bars, vertical: 5 mV, horizontal: 5 ms. From Dietz and Manahan-Vaughan, 2017. **(B)** Novel Exploration of a spatial configuration of odors (top) also promotes the expression of LTD. The graph shows the expression of LTD when wLFS (applied to SC-CA1 synapses) was combined with *de novo* exposure to different odors that emanated from holes in the floor of the chamber. Re-exposure to the same odors in the same spatial locations ca. 1 week after the first exposure failed to induced LTD. From André and Manahan-Vaughan (2013). **(C)** Exploration of spatial configurations of novel visual items promotes LTD (top). Graph: novel exploration of spatially distributed visual items during wLFS of SC-CA1 synapses enables LTD. Re-exposure to the same items in the same spatial configuration during wLFS 1 week later results in STD, whereas exposure to a new spatial configuration of the visual items results in LTD that lasts for over 24 h. Inset: analogs show examples of fEPSPs recorded prior to wLFS (left column), 5 min (middle column) and 24 h (right column) after wLFS in animals that engaged in novel visuospatial cue exploration (top row), animals that experienced re-exposure to the cues (middle row) and animals that were exposed to a novel visuospatial cue configuration (bottom row). Scale bars, vertical: 5 mV, horizontal: 5 ms. From Kemp and Manahan-Vaughan (2004). Cartoons **(A–C)** were modified from: Manahan-Vaughan (2018b).

The facilitation of hippocampal LTD by visuospatial item-place learning does not only occur when an animal navigates around and explores objects in the physical domain. The viewing of item-place constellations on a computer screen by inert rats also enables LTD (Kemp and Manahan-Vaughan, 2012), suggesting that this phenomenon involves cognitive processing. In line with this, others have shown that inhibition of calcineurin, a key molecular step in the expression of LTD, prevents episodic-like learning in rodents (Zeng et al., 2001). Indeed a comparison of the viewing of visuospatial item constellations at the level of event-related potentials by humans and rats has revealed striking common denominators, including structures such as the posterior parietal cortex (Hauser et al., 2019). The induction of hippocampal LTD by item-place experience is not restricted to the visuospatial domain, however. Spatial configurations of olfactory (André and Manahan-Vaughan, 2013) and auditory items (Dietz and Manahan-Vaughan, 2017) also facilitate the expression of hippocampal LTD (Figures 2A–C).

In contrast to LTP that is expressed, albeit in an input-specific manner, in a widespread distribution across hippocampal subfields in response to a novel allocentric experience (Straube et al., 2003; Kemp and Manahan-Vaughan, 2004, 2008b; Hagena and Manahan-Vaughan, 2011), LTD expression is synaptic subcompartment-specific and this property, in turn, is mediated by specific kinds of item-place experience. Thus, if spatial content pertains to subtle features of the environment that can only be discovered if the animal is physically beside them, LTD is expressed in Schaffer collateral-CA1 synapses (Manahan-Vaughan and Braunewell, 1999; Kemp and Manahan-Vaughan, 2004, 2008b), or commissural-associational-CA3 synapses (Hagena and Manahan-Vaughan, 2011). These features can be visual, olfactory or auditory (André and Manahan-Vaughan, 2013; Dietz and Manahan-Vaughan, 2017; Figure 2). But if the environmental features are large and visible from afar, LTD at perforant path-dentate gyrus, and mossy fiber-CA3 synapses is induced (Kemp and Manahan-Vaughan, 2008b; Hagena and Manahan-Vaughan, 2011). Exposing animals to a novel environment with both distinct novel allocentric and novel content cues triggers hippocampal LTP that segues into LTD (Manahan-Vaughan and Braunewell, 1999). This finding suggests that hippocampal subfields and their synaptic subcompartments are highly specialized with regard to the functional expression of LTD in response to different kinds of item-place experience.

### The Link Between Long-Term Depression, Spatial Information Updating and Prevention of Experience Generalization

However, it is not only novel item-place constellations that promote the expression of LTD: modifications of spatial configurations conducted by moving familiar items into unfamiliar spatial positions also triggers LTD (Manahan-Vaughan and Braunewell, 1999; Kemp and Manahan-Vaughan, 2004, 2008b,^2012^; Goh and Manahan-Vaughan, 2013c). This takes place in perforant path-dentate gyrus, and mossy fiber-CA1, synapses when a known spatial arrangement of large landmark features is changed without altering the ostensible content of the spatial environment (Kemp and Manahan-Vaughan, 2008b; Hagena and Manahan-Vaughan, 2011). LTD, at Schaffer collateral-CA1 synapses and commissural associational-CA3 synapses, is also triggered when subtle, less obviously visible visuospatial configurations of familiar items are altered (Manahan-Vaughan and Braunewell, 1999; Kemp and Manahan-Vaughan, 2004; Goh and Manahan-Vaughan, 2013c). Taken together, these findings indicate that LTD supports the fine-tuning of experience-dependent storage of spatial knowledge in a hippocampal neuronal network, that relates, in turn, to the postulated role of the different hippocampal subfields in the acquisition of knowledge about orientational and content features of space (Jacobs and Schenk, 2003).

Further evidence for a role for LTD in information updating comes from studies of reversal and extinction learning. During reversal learning, rodents typically learn the (constant) location of a hidden platform over a series of training trials. Multiple trials result in the animals acquiring an accurate spatial representation of the location of the platform relative to allocentric cues. One can assess reversal learning, and thereby behavioral flexibility, by then changing the location of the platform and examining how rapidly the animal builds a new representation (or continues to look for the platform where it was previously located). Inhibition of LTD prevents reversal learning (Nicholls et al., 2008; Kim et al., 2011, 2017; Dong et al., 2013; Mills et al., 2014) and also prevents extinction learning (Kim et al., 2017), whereby due to changing contingencies a previous behavior should no longer be executed.

These properties of LTD raise the possibility that by serving as a cellular mechanism for representational updating, LTD may also circumvent that very similar experiences become generalized. This not only would serve as an invaluable mechanism to ensure the integrity and reliability of similar memories, but could be expected to support pattern separation, and prevent the generalization of traumatic experience. Evidence for this has been provided by studies that reported on the one hand, that LTD subserves the temporal compartmentalization of acquired memories (Cui et al., 2013) and also that improving LTD prevents the generalization of fear memory (Cao et al., 2021).

**FIGURE 3:**
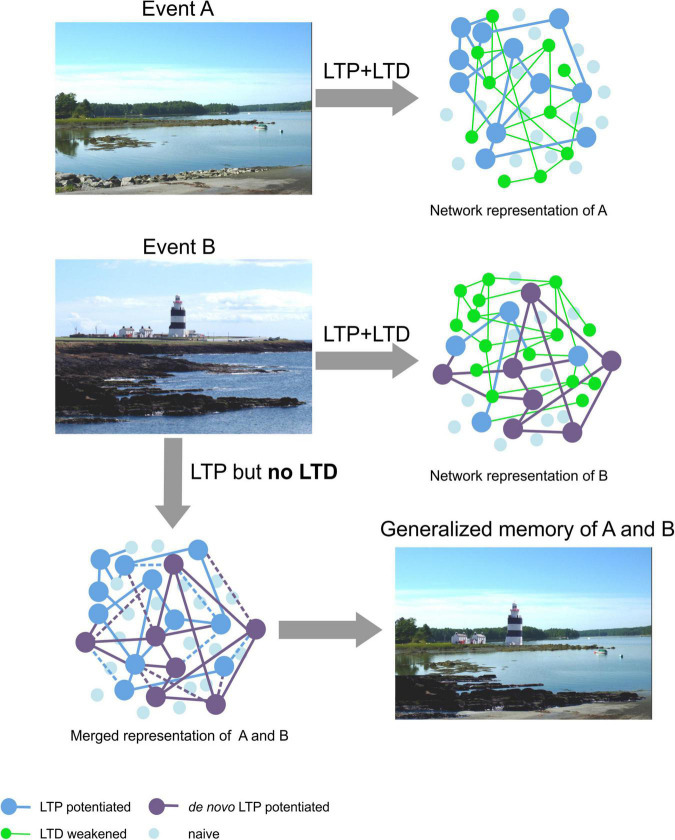
Hypothetical schema of the proposed role for LTD in enabling discriminable spatial representations. Top: The upper photo (Event A, left) is of a landscape near Damariscotta in Maine, United States. By means of LTP the general schema of this landscape is presumably obtained (large dark blue dots, right) (Kemp and Manahan-Vaughan, 2007; Manahan-Vaughan, 2017). Content details are retained by means of LTD (Kemp and Manahan-Vaughan, 2008a; Manahan-Vaughan, 2017) that serves to eliminate weakly potentiated synapses (green dots), or weaken communications between synapses. By this means a robust representation is obtained. Middle: The photo (left) is of Hook Head in Ireland (Event B). When we acquire new memories we are very likely to use blueprints of past memories of similar experiences. Thus, elements of a previously stored neuronal and synaptic network can be re-used as a framework for, in this case, the promontory-like features of the scene, the water inlets and the general global composition of the landscape encoded in Event A. LTD serves to remove superfluous elements, of the new representation compared to the Maine landscape (the asphalt element in the foreground, the trees lining the horizon). *De novo* LTP is likely to support the retention of new general features of the landscape (large purple dots, right) (Kemp and Manahan-Vaughan, 2004; Manahan-Vaughan, 2018a) and LTD contributes to information encoding through the inclusion of content details such as the houses and the lighthouse (Kemp and Manahan-Vaughan, 2008a; Manahan-Vaughan, 2017, 2018a). Where LTP and LTD work together, LTD serves to modify the new network, thereby enabling pattern separation (Manahan-Vaughan, 2018a; Collitti-Klausnitzer et al., 2021). Bottom left: In the absence of the refinement of signal-to-noise ratios and suppression of redundant synaptic connections, in the new representation by means of LTD, the former potentiated network merges with the new network and the memory of both experiences becomes generalized into one representation (bottom right). Photos: D. Manahan-Vaughan.

We propose that LTD strengthens the robustness of stored experience by pruning away synapses that are weakly integrated into a synaptic network that stores a specific experience. LTD also temporally and physically constrains LTP into specific synaptic and dendritic subcompartments thereby preventing a “seepage” and binding of one discrete memory into another either similar, or recently acquired memory. By this means, erroneous associations are avoided and the integrity of a stored experience is secured. This process can be considered an integral element of pattern separation whereby very similar experiences can be disambiguated from one another. Assuming that LTP creates the memory engram by strengthening selected synapses within a network, and assuming that similar experiences may recruit information storage in overlapping synaptic circuitry, LTD thereby may serve to sharpen the resolution of these representations by minimizing overlap. We propose that without LTD, memory generalization can occur that confounds disambiguation of similar experiences (Figure 3). This possibility is supported by findings that animals with impairments in LTD quickly forget conditioned taste aversion and consume more of the conditioned substance than controls (Toyoda et al., 2020), are unable to learn the platform location in a water maze and show long-term memory deficits in a Barnes maze (Rocchetti et al., 2015). They also display enhanced freezing behavior, in the absence of foot shock, weeks after context-dependent conditioning (Navarrete et al., 2019). This is consistent with behavioral generalization associated with impoverishment of pattern separation. The ability of LTD to refine synaptic networks generated by means of LTP may be of particular importance in contextual learning. Although generalization of memory may also involve depotentiation of LTP at potentiated synapses that creates an instability of the memory representation (Richards and Frankland, 2017; Robertson, 2018), as mentioned above, evidence for a role for LTD in the protection against memory generalization has also been reported (Cui et al., 2013). These truly are fascinating possibilities, and the question arises as to how they could be mechanistically and anatomically realized.

## Physical Processes Underlying Long-Term Depression Contributions to Memory

### Hippocampal Long-Term Depression Triggers Gene Transcription

Induction of hippocampal synaptic plasticity results in the activation of members of the Fos, Jun, Krox, and Arc families of immediate early genes (IEGs; Manahan-Vaughan, 2017). The temporal pattern and distribution of neuronal expression of IEGs is determined by whether LTP, or LTD, is induced (Yilmaz-Rastoder et al., 2011; Hoang et al., 2021) and also depends on the kind of behavioral learning task implemented (Miyashita et al., 2009; Pevzner et al., 2012), or form of learning-facilitated synaptic plasticity that was instigated (Hoang et al., 2021; Table 2). The persistent (> 24 h) expression of hippocampal LTD requires protein translation in the CA1 region (Manahan-Vaughan, 2000), at commissural associational-CA3 and mossy fiber-CA3 synapses (Hagena and Manahan-Vaughan, 2013), but not in the dentate gyrus (Pöschel and Manahan-Vaughan, 2007). This latter finding might be explained by the fact that electrophysiological recordings were performed from the upper (suprapyramidal) layer of the dentate gyrus in the study by Pöschel and Manahan-Vaughan (2007), whereas more recent studies of gene encoding triggered by LTD have identified the lower (infrapyramidal) layer as being the site of somatic immediate early gene expression triggered by learning-facilitation of LTD (Hoang et al., 2021). IEGs that have either been reported to be essential for learning–mediated LTD, or are triggered by it comprise c-Fos, Homer1a and Arc (Kemp et al., 2013; Hoang et al., 2021). Blocking of c-Fos mRNA prevents learning-mediated LTD facilitation (Kemp et al., 2013), and slices from Arc knockout mice show impaired LTD (Plath et al., 2006), indicating causal contributions of c-Fos and Arc to LTD processes. In a non-behavioral setting, hippocampal LTD (> 24 h) is typically triggered by low frequency stimulation (LFS) of hippocampal afferents. In a behavioral setting LTD is enabled by coupling weak afferent stimulation with a spatial learning event, referred to as learning-facilitated LTD (Manahan-Vaughan, 2018a,b). Homer1a expression is triggered by LFS-induced LTD in all hippocampal regions and by learning-facilitated LTD (landmark exploration) in the CA3 and DG subfields (Hoang et al., 2021; Table 2). Expression is both task and synaptic subcompartment-specific and also relatively sparse. The sparseness depends on the particular IEG, brain region and the specific experience. Experience-dependent expression can be as low as 4–8% in the dentate gyrus, but can also reach levels up to 30% in the cornus ammonis (Hoang et al., 2018, 2021). It should be noted that a sparse population of c-Fos positive neurons in DG (e.g., 6%) may be sufficient to recall the encoded behavior (Liu et al., 2012). Thus sparse and specific IEG expression may enable the selective modulation of discrete hippocampal circuitry. In line with this it was recently shown that Fos expression that was triggered by prolonged exposure of mice to enriched spatial content, results in a differentiated modulation of the inhibitory output of specific populations of interneurons in the hippocampal CA1 region (Yap et al., 2021). By this means, discrete anatomical subcompartments of pyramidal cells can be modulated. This may support the modification and “pruning” of synaptic networks such that stable spatial representations result.

**TABLE 2:** Overview of subcompartment-specific cell-nucleus expression of immediate early genes triggered by either different kinds of spatial learning or by task-specific facilitation of hippocampal LTP or LTD.

Synaptic plasticity facilitated by new spatial exploration or induced by stimulation	dCA1	pCA1	dCA3	pCA3	uDG	lDG
LTP, empty holeboard	↑H1a^2^	↑H1a^2^	↑H1a^2^	↑H1a^2^	↑H1a^2^	↑H1a^2^
LTD, small objects	↑Arc^1^	– Arc^1^	– Arc^1^	↑Arc^1^	– Arc^1^	– Arc^1^
	↑ c-Fos^4^*					
LTD, landmarks	– Arc^1^, H1a^2^	– Arc^1^, H1a^2^	–H1a^1,2^	↑H1a^1,2^	–Arc^1^, H1a^1,2^	↑Arc^1^, H1a^1,2^
LTP induced by HFS	↑ H1a^2^	↑ H1a^2^	↑ H1a^2^	↑ H1a^2^	↑ H1a^2^	↑ H1a^2^
	↑ Arc^3^**					
LTD induced by LFS	↑ H1a^2^	↑ H1a^2^	↑ H1a^2^	↑ H1a^2^	↑ H1a^2^	↑ H1a^2^
	↓↑Arc^3^**					

*The expression of c-Fos was analyzed in the whole hippocampus without subdifferentiation.

**Arc expression was analyzed only in the dorsal CA1, without differentiation between proximal and distal subdivisions.

*The abbreviations correspond to the dorsal CA1 (dCA1), proximal CA1 (pCA1), dorsal CA3 (dCA3), proximal CA3 (pCA3), the upper (suprapyramidal) blade of the DG (uDG), and the lower (infrapyramidal) blade of the DG (lDG).*

*An arrow indicates that a significant increase in IEG expression [cFos, Arc or Homer1a (H1a)] was triggered by the event.*

*A dash signifies that no change occurred compared to naïve controls.*

*What is striking is that LTP that is facilitated by de novo exposure to unfamiliar space results in IEG expression in neuronal populations across all hippocampal subfields and subcompartments.*

*By contrast, LTD that is facilitated by learning results in an expression-pattern that is tightly dependent upon the kind of spatial content learning conducted.*

*The data summarized in the table were reported in ^1^Hoang et al. (2018), ^2^Hoang et al. (2021), ^3^Yilmaz-Rastoder et al. (2011), and ^4^Kemp et al. (2013).*

Studies using fluorescence *in situ* hybridization to study cell compartment-specific expression of IEGs in neuronal nuclei hint that LTD may indeed modify neuronal networks. Thus, whereas the enablement of LTP by spatial learning results in IEG expression throughout all hippocampal subfields, LTD facilitation by spatial learning results in a differentiated and hippocampal subfield-specific elevation of nuclear IEG expression (Hoang et al., 2018). We believe that this effect is functionally highly meaningful. Consider the abovementioned findings of Yap et al. (2021) who reported that spatial learning results in discrete IEG-dependent modulation of the output of hippocampal interneurons: We recently reported that identical stimulation patterns, when applied to the lateral or medial entorhinal cortex inputs to the dentate gyrus, produce radically different synaptic plasticity outcomes within the same approximate population of granule cells in the dentate gyrus (Collitti-Klausnitzer et al., 2021). Most striking is the preference of the medial perforant path-dentate gyrus synapses to express LTP, and of the lateral perforant path-dentate gyrus to express LTD. Whereas the lateral perforant path provides information from the lateral entorhinal cortex to the hippocampus about the animal’s egocentric relationship to features of space, the medial perforant path provides information about the animal’s allocentric position in space (Lisman, 2007; van Strien et al., 2009; van Cauter et al., 2013; Wang et al., 2018, 2020). Interneurons in the dentate gyrus allow very discrete control of dendritic and axonal compartments (Houser, 2007). IEG expression driven by the experience-dependent induction of LTD in the dentate gyrus may thus enable highly specific modifications of a synaptic and neuronal ensemble, such that spatial information about egocentric and allocentric experience can be disambiguated.

### Both Learning and Long-Term Depression Promote Structural Plasticity

Dendritic spines are highly dynamic and can change their density, morphology and volume in response to neuronal activity and experience (for reviews see Kasai et al., 2010; Fu and Zuo, 2011; Rochefort and Konnerth, 2012; Gipson and Olive, 2017; Chidambaram et al., 2019; Runge et al., 2020). For this reason, they have been proposed to be the site of memory storage in the brain (Nishiyama and Yasuda, 2015; Segal, 2017). Stimulation of single spines induces their enlargement (Maletic-Savatic et al., 1999; Matsuzaki et al., 2004; Nishiyama and Yasuda, 2015) that is, in turn, associated with increased α-amino-3-hydroxy-5-methyl-4-isoxazolepropionic acid receptor (AMPAR) currents as well as LTP (Matsuzaki et al., 2004). Induction of LTP, conversely, triggers synaptogenesis (Oe et al., 2013; Hasegawa et al., 2015). Particularly, LTP induction (by high frequency stimulation, HFS, or theta burst stimulation, TBS) in CA1 dendrites induces enlargement or *de novo* growth of spines (Engert and Bonhoeffer, 1999; Matsuzaki et al., 2004; Nägerl et al., 2004, 2007) that can form mature synapses mostly with pre-existing synaptic boutons (Toni et al., 1999; Nägerl et al., 2007). Moreover, LTD, induced by LFS, in the CA1 region has been associated with spine shrinkage or retraction (Nägerl et al., 2004; Zhou et al., 2004), as well as increased synaptic bouton turnover and decreased volume of boutons associated with retracted spines (Becker et al., 2008).

Could synaptic plasticity, and most specifically LTD, serve as a cellular mechanism whereby synaptic remodeling, in conjunction with long-term memory formation, is enabled? A causal link between LTD and spine structural plasticity is supported by several experimental findings (Matsuzaki et al., 2001, 2004; Becker et al., 2008; Kwon and Sabatini, 2011; Wiegert and Oertner, 2013; Bosch et al., 2014; for reviews see Bosch and Hayashi, 2012; Nishiyama and Yasuda, 2015; Suratkal et al., 2021). A further link between synaptic plasticity and spine remodeling is provided by the postsynaptic actin cytoskeleton that plays a major role in structural and functional aspects of dendritic spines (Gipson and Olive, 2017; Runge et al., 2020; Suratkal et al., 2021). It undergoes a constant turnover of polymerized filamentous (F)-actin, or depolymerized globular (G)-actin. It was shown that HFS of the hippocampus switches the equilibrium toward F-actin, whereas LFS increases G-actin (Okamoto et al., 2004). Importantly, these frequency-dependent modulations of actin polymerization/de-polymerization occur concomitantly with spine enlargement (in case of HFS) and spine shrinkage (in case of LFS). Thus, induction of synaptic plasticity is accompanied by actin modifications underlying dendritic spine remodeling, further supporting the link between these two processes. These findings also suggest that spine remodeling and synaptic plasticity may share molecular mechanisms.

Indeed, structural and functional synaptic plasticity share a common denominator in terms of the time sequence of the synaptic changes upon activation (Nishiyama and Yasuda, 2015) and also share molecular mechanisms (Table 3). The actin cytoskeleton grows within minutes after LTP induction, with actin and actin-binding proteins (such as cofilin) accumulating in the spine. The actin cytoskeleton is first rapidly remodeled and subsequently stabilized (Bosch et al., 2014). The actin modification by actin-binding proteins contributes to enhanced stabilization (decreased de-polymerization) of F-actin which by a continuous actin polymerization possibly leads to spine expansion (Bosch and Hayashi, 2012; Nishiyama, 2019). After a few hours, the postsynaptic density (PSD) size increases, followed also by growth of presynaptic terminals (Bosch et al., 2014; Meyer et al., 2014; Nishiyama and Yasuda, 2015). Interestingly, the increase in PSD size, in PSD components (such as PSD-95) and in the presynaptic bouton re-establishes the correlation of these components to spine volume (that increased shortly after stimulation) and allows stabilization of the enlarged synapse (Meyer et al., 2014). If one or more of these subsynaptic components do not increase in size, the spine volume and synapse size return to their initial state.

**TABLE 3:** Molecular pathways of LTP/spine enlargement and LTD/spine shrinkage.

Molecule	LTP/spine enlargement	LTD/spine shrinkage
NMDAR GABA-R (involved in LTD) (mGluR—shrinkage of large spines)	Ca^2+^ increase → CaMKII activation	Ca^2+^ concentration regulation, calcineurin activation
CaMKII	small GTPase activation; AMPAR regulation; NMDAR stabilization;	AMPAR regulation
Rac GTPase	regulation of actin binding proteins, Arp2/3 and cofilin, *via* WAVE and PAK-LIMK pathways, respectively; AMPAR regulation	
Cdc42	regulation of actin binding proteins, Arp2/3 and cofilin, *via* WASP and PAK-LIMK pathways, respectively; support of hippocampal LTP	
Calcineurin		actin depolymerization e.g., *via* cofilin; AMPAR dephosphorylation
p38 MAPK		actin depolymerization through activation of cofilin *via* MAPK-activated protein kinase 2; AMPAR endocytosis

*Overview of some molecules that exert effects on both structural and functional plasticity (left column).*

*The middle column indicates the role of these molecules in LTP and dendritic spine enlargement, while the right column describes the effects of molecules involved in LTD and spine shrinkage [based on Okamoto et al. (2004); Holbro et al. (2009); Baumgärtel and Mansuy (2012); Bosch and Hayashi (2012); Coultrap and Bayer (2012); Hayama et al. (2013); Oh et al. (2013); Kim et al. (2014); Nishiyama and Yasuda (2015); Borovac et al. (2018); Woolfrey et al. (2018); Zhang et al. (2018); Nishiyama (2019); Stein and Zito (2019); Costa et al. (2020); Runge et al. (2020); Stein et al. (2020); and Suratkal et al. (2021)].*

*AMPAR, α-amino-3-hydroxy-5-methyl-4-isoxazolepropionic acid receptor; Arp, actin-related protein; CaMKII, calcium calmodulin kinase II; Cdc42, cell division control protein-42 homolog; GABA-R, gamma-aminobutyric acid receptor; GTP, guanosine triphosphate; LIMK, LIM kinase, MAPK, mitogen-activated protein kinase; mGluR, metabotropic glutamate receptor; NMDAR, N-Methyl-D-aspartate receptor; PAK, p21-activated kinase; WASP, Wiskott-Aldrich syndrome protein; WAVE, WASP family verprolin homologous (protein).*

Hippocampal LTD includes pre-and postsynaptic components (Pöschel and Stanton, 2007). LTD that occurs under circumstances of spatial learning mediates AMPAR endocytosis, suggesting that synapse-specific modifications take place (Ge et al., 2010; Ashby et al., 2021). In line with this, it has been shown that following induction of hippocampal LTD, pre-and postsynaptic structures become segregated (Bastrikova et al., 2008). Furthermore, after induction of hippocampal LTD, depressed synapses are eliminated from hippocampal circuitry (Hasegawa et al., 2015) and effects are N-methyl-D-aspartate receptor (NMDAR)-dependent (Wiegert and Oertner, 2013). Strikingly, this process is accompanied by a stabilization of the persistency of the retained fraction of spines on affected dendrites (Wiegert and Oertner, 2013). Moreover, whereas LTP increases synaptic stability at the level of dendritic spines, LTD weakens it, but LTD is not able to destabilize potently induced LTP (Wiegert et al., 2018). This latter finding aligns with our own findings that the potent induction of hippocampal LTP using strong HFS protocols induces higher levels and more widespread distribution of nuclear IEG expression than LTP induced by behavioral learning (Hoang et al., 2021). Others have reported that LTP is induced by fear conditioning (Whitlock et al., 2006; Subramaniyan et al., 2021) and that fear conditioning is associated with generalization of fear memory to a non-threatening environment (Liu et al., 2012). This raises the possibility that potently induced LTP may be associated with the generalization of memories, and that this may occur because it is invulnerable to modification by LTD.

The stabilization of new spines has also been linked to synaptic plasticity mechanisms involving calcium/calmodulin-dependent protein kinase II (CaMKII; Wilbrecht et al., 2010. Furthermore, changes in the number and persistency of synaptic spines is also driven by sensory experience (Holtmaat et al., 2006). Thus, both neuronal activity and sensory experience lead to spine remodeling (Rochefort and Konnerth, 2012); but how are these processes connected to learning and behavior? Bidirectional spine changes have been demonstrated in *in vivo* studies (Fu and Zuo, 2011; Rochefort and Konnerth, 2012). For example, although motor skill learning initially triggers spine formation, and the fraction of new spines that emerges correlates with task acquisition performance, memory retention after training over a period of several days correlates with the degree of spine *elimination* (Xu et al., 2009; Yang et al., 2009). Auditory fear conditioning and extinction learning are also associated with spine elimination and new spine formation in the cortex (Lai et al., 2012, 2018; Yang et al., 2016). Others have reported similar results in the hippocampus following contextual fear conditioning, whereby spine elimination (measured 24 h after fear conditioning) occurred particularly on hippocampal neurons that were activated during learning (Sanders et al., 2012). Thus, spine remodeling appears to comprise an important building block of memory circuits. Furthermore, structural changes in dendritic spines appear highly specifically in conjunction with cued learning, and correlate with memory performance.

Taken together, it is possible that experience-dependent LTD mediates synapse elimination and pruning of “LTP circuitry” at the level of spine insertion and enlargement, thereby refining the resolution, stability and integrity of synaptic networks. We propose that the benefit to synaptic circuitry, that is occupied with long- term experience-dependent information storage, is an increase in signal-to-noise ratios of those networks involved in retaining memories, such that similar experiences can be more easily disambiguated from one another.

### Temporal and Physical Constraint of Long-Term Potentiation by Long-Term Depression

From a temporal point of view, LTP is induced very rapidly (Gustafsson and Wigström, 1990), whereas LTD requires minutes to emerge (Dudek and Bear, 1993; Manahan-Vaughan and Braunewell, 1999; Klausnitzer and Manahan-Vaughan, 2008). This property may enable LTD to refine a recently acquired memory and help optimize accurate memory retention. In line with this possibility, LTD has been implicated in spatial memory consolidation (Ge et al., 2010), and pharmacological prevention of LTD prevents both the acquisition of an accurate memory of spatial experience and learning-facilitation of LTD (Kemp and Manahan-Vaughan, 2008a; Popkirov and Manahan-Vaughan, 2011; Hagena and Manahan-Vaughan, 2012; Lemon and Manahan-Vaughan, 2012; Hagena et al., 2016; Dietz and Manahan-Vaughan, 2017). Under circumstances where a spatial paradigm, used to trigger LTP, was combined with spatial content elements that enable LTD, it became apparent that an initial potentiation of synapses was followed minutes later by LTD (Manahan-Vaughan and Braunewell, 1999) suggesting that LTP and LTD are processes that can occur concomitantly in the same synaptic population. This property has, in fact, been reported within the entorhinal cortex, where it was shown that LTP and LTD can be expressed in the basal and apical dendritic compartments of the same pyramidal cell population (Solger et al., 2004). The same property was later reported in the hippocampus (Pavlowsky and Alarcon, 2012). These studies show that LTP and LTD can be triggered within the same dendritic compartment, but their dual manifestation is spatially regulated and activity-dependent.

Coincident expression of LTP and LTD has also been reported at hippocampal synapses. Here, for example induction of homosynaptic LTP results in heterosynaptic LTD (Stanton and Sejnowski, 1989). But coincident activity in the (subsequently) depressed synapse must occur at the time-point of the LTP event in order for heterosynaptic LTD to occur (Abraham et al., 2007), suggesting that heterosynaptic LTD is not a passive side effect of homosynaptic LTP induction, but is actually an active part of network modification. Moreover, the degree of heterosynaptic interactions between LTP and LTD is determined by the degree of overlap of the terminal fields of afferent inputs, and thereby of dendritic fields (White et al., 1990), These observations fit well with the possibility that one of the tasks of LTD is to improve signal-to-noise ratios during information encoding by means of LTP, and with the likelihood that LTD can constrain the physical distribution of LTP in a synaptic network.

Earlier in this article, we described how different components of spatial learning can result in the synaptic subcompartment-specific expression of LTD in the hippocampus. Specific afferent inputs corresponding to the dorsal and ventral streams (Mishkin et al., 1983) can determine to some extent which kind of information content is delivered to specific hippocampal subfields (Amaral and Witter, 1989; Burke et al., 2011; Sauvage et al., 2013; Hoang et al., 2018). But a further disambiguation of this information needs to take place at the level of the dendritic field, so that experience-dependent encoding at the level of LTP and LTD can take place, assuming these are the primary determinants of disambiguated spatial information storage. In this context, it has been reported that, in the CA1 region, both LTP and LTD induced by patterned afferent stimulation (e.g., HFS at 10 Hz, LFS at 1 Hz) is greater in magnitude in the dendritic compartment that is distal to the pyramidal cell layer, compared to plasticity that is expressed proximally to the pyramidal cell layer (Aihara et al., 2005). By contrast, if a stochastic-like stimulation pattern was used the LTP expression pattern remained the same (distal > proximal) with its magnitude being determined by the stimulation frequency used. By contrast, LTD expression occurred in a uniformal distributed manner across all dendritic subcompartments (distal = proximal) (Aihara et al., 2005). Furthermore, it has been reported that LTD that is expressed in the distal dendrites persists for longer than LTD expression in proximal dendrites (Ramachandran et al., 2015). This suggests that depending on the afferent input and the dendritic subcompartment in which LTD is expressed, the influence of LTD on expression patterns of LTP will vary. This influence also extends into the domain of metaplasticity, through which the prior history of activity-dependent experience of a synapse influences subsequent plasticity events (Abraham, 2008): prior induction of LTD reduces the magnitude of a subsequent induction of LTP (and vice versa), and simultaneous induction of LTP and LTD reduces the magnitude of LTP expressed (Pavlowsky and Alarcon, 2012). These processes suggest that LTD can temporally and physically constrain LTP into discrete synaptic subcompartments. One consequence of this process would be the limitation of the building of associations with other recently acquired representations, which at a plasticity level could be expected to prevent processes such as synaptic tagging (Frey and Morris, 1998). At a behavioral level this process would prevent generalization of memories and serve to optimize pattern separation. Behavioral evidence for this derives from studies that show that enhancement of LTD reduces fear memory generalization (Cao et al., 2021) and that inhibition of LTD prevents both item-place recognition memory (Dong et al., 2012; Goh and Manahan-Vaughan, 2013b; Kim et al., 2017; Ledonne et al., 2018) and spatial information updating (Kemp and Manahan-Vaughan, 2008b; Popkirov and Manahan-Vaughan, 2011; Lemon and Manahan-Vaughan, 2012; Dong et al., 2013).

## Conclusion

Findings derived from the anatomical and cellular scrutiny of neuronal changes in learning and plasticity events, long with studies that interlink hippocampal LTP and LTD with spatial learning, indicate the interdependence of these processes and provide a plausible explanation as to how learning can be related to both structural and functional plasticity in the brain. Furthermore, the close interrelationship of these processes provides fascinating insights as to how persistent increases and decreases of synaptic efficacy are implemented in the brain to support memory formation, on both structural and functional levels.

Synaptic connections between neurons are considered to be the major site of information storage in the brain. Consequently, sensory experience and learning elicit physiological and structural modifications of synapses that are the neuronal substrate correlated with this experience. Meticulous research has provided us with substantial knowledge about synaptic modifications related to learning and memory. The size or number of dendritic spines can both increase and decrease in response to learning. Increase in spine formation and shrinkage of spines have been associated with LTP and LTD, respectively. Furthermore, LTD has been shown to modulate the magnitude of LTP and to constrain its expression both on physical (dendritic subcompartment) and temporal (metaplastic) levels. Behavioral studies that integrate the scrutiny of memory acquisition and retention have demonstrated a role for LTD in item-place memory, spatial content learning and representation updating. Furthermore, studies using pharmacology or transgenic manipulations infer a role for LTD in preventing memory generalization, pattern separation and optimization of the integrity of memories of spatial experience. Taken together, current evidence suggests that hippocampal LTD uniquely contributes to spatial learning and memory, most particularly in the support of the acquisition, updating and unadulterated long-term memory of spatial content.

## Author Contributions

Both authors listed have made a substantial, direct, and intellectual contribution to the work, and approved it for publication.

## Conflict of Interest

The authors declare that the research was conducted in the absence of any commercial or financial relationships that could be construed as a potential conflict of interest.

## Publisher’s Note

All claims expressed in this article are solely those of the authors and do not necessarily represent those of their affiliated organizations, or those of the publisher, the editors and the reviewers. Any product that may be evaluated in this article, or claim that may be made by its manufacturer, is not guaranteed or endorsed by the publisher.
